# Protocol for the saMS trial (supportive adjustment for multiple sclerosis): a randomized controlled trial comparing cognitive behavioral therapy to supportive listening for adjustment to multiple sclerosis

**DOI:** 10.1186/1471-2377-9-45

**Published:** 2009-08-23

**Authors:** Rona Moss-Morris, Laura Dennison, Lucy Yardley, Sabine Landau, Suzanne Roche, Paul McCrone, Trudie Chalder

**Affiliations:** 1 School of Psychology, University of Southampton, Highfield Campus, Southampton, SO17 1BJ, UK; 2 Department of Biostatistics, Institute of Psychiatry, King's College London, De Crespigny Park, London, SE5 8AF, UK; 3 Chronic Fatigue Unit, Maudsley Hospital, Denmark Hill, London, SE5 9RS, UK; 4 Health Service and Population Research Department, Institute of Psychiatry, King's College London, De Crespigny Park, London, SE5 8AF, UK; 5 Department of Psychological Medicine, Institute of Psychiatry, King's College London, Weston Education Centre, Cutcombe Road, London, SE5 9RJ, UK

## Abstract

**Background:**

Multiple Sclerosis (MS) is an incurable, chronic, potentially progressive and unpredictable disease of the central nervous system. The disease produces a range of unpleasant and debilitating symptoms, which can have a profound impact including disrupting activities of daily living, employment, income, relationships, social and leisure activities, and life goals. Adjusting to the illness is therefore particularly challenging. This trial tests the effectiveness of a Cognitive Behavioural intervention compared to Supportive Listening to assist adjustment in the early stages of MS.

**Methods/Design:**

This is a two arm randomized multi-centre parallel group controlled trial. 122 consenting participants who meet eligibility criteria will be randomly allocated to receive either Cognitive Behavioral Therapy or Supportive Listening. Eight one hour sessions of therapy (delivered over a period of 10 weeks) will be delivered by general nurses trained in both treatments. Self-report questionnaire data will be collected at baseline (0 weeks), mid-therapy (week 5 of therapy), post-therapy (15 weeks) and at six months (26 weeks) and twelve months (52 weeks) follow-up. Primary outcomes are distress and MS-related social and role impairment at twelve month follow-up. Analysis will also consider predictors and mechanisms of change during therapy. In-depth interviews to examine participants' experiences of the interventions will be conducted with a purposively sampled sub-set of the trial participants. An economic analysis will also take place.

**Discussion:**

This trial is distinctive in its aims in that it aids adjustment to MS in a broad sense. It is not a treatment specifically for depression. Use of nurses as therapists makes the interventions potentially viable in terms of being rolled out in the NHS. The trial benefits from incorporating patient input in the development and evaluation stages. The trial will provide important information about the efficacy, cost-effectiveness and acceptability of the interventions as well as mechanisms of psychosocial adjustment.

**Trial registration:**

Current Controlled Trials ISRCTN91377356

## Background

### Psychological adjustment to Multiple Sclerosis

Multiple Sclerosis (MS) is an incurable, chronic and unpredictable disease of the central nervous system. The disease is characterized by the destruction of the myelin sheath surrounding the nerves resulting in the formation of plaques. These plaques disrupt the transmission of nerve impulses leading to the symptoms of the illness which include, but are not limited to, spasticity, loss of balance and co-ordination, blurred or double vision, blindness, numbness, speech distortions, bladder and bowel problems, fatigue, pain and cognitive dysfunction [1]. Plaques can occur in a variety of sites resulting in substantial variation in the type and nature of the symptoms across individuals. The course of the illness is also highly variable and unpredictable [1]. The majority have either a relapsing-remitting or a relapsing-progressive course. Patients experience periods of partial or total remission where the illness is inactive, interspersed with symptom relapses. MS can also have a chronic-progressive course, in which there is a progressive worsening of symptoms and disability. Patients may be initially diagnosed with one type of MS, but over time progress to another.

MS is thought to affect more than 2.5 million people worldwide and around 400,000 people in the United States and 85,000 people in the United Kingdom currently live with the disease [2,3]. The illness is more common in females than males. The cause of the illness is largely unknown and there is currently no cure. Treatment focuses on the management of the patient's symptoms and reducing the number and severity of relapses.

Individuals who have MS are faced with uncertainty about the future, unpleasant and unpredictable symptoms, treatment regimes and drug side effects. MS can have profound consequences including disruptions to life goals, employment, income, relationships, social and leisure activities and activities of daily living. Therefore it is not surprising that it poses multiple challenges for psychological adjustment. A large body of empirical literature attests to poor adjustment outcomes in MS including elevated rates of depressive symptomology or distress [4-7], increased anxiety [5,8], poor subjective well-being and quality of life [5,9], and social role and relationship difficulties [4,10]. On the other hand, research and clinical experience suggests that a substantial proportion of people with MS manage to adapt well to living with the illness [11,12].

Illness factors such as the extent of neurological disability, symptom severity, remission status and length of illness can influence levels of adjustment or a sense of well-being and quality of life [13,14]. However, psychological factors can be as important in predicting and explaining individual differences in adjustment [14,15]. A systematic review conducted as pilot work for this trial demonstrated that a range of psychological factors were associated with adjustment outcomes in MS [16]. For instance, people reporting high levels of perceived social support showed better adjustment [17,18], as did individuals with high levels of optimism and hope [19,20] and those who engaged in health behaviours such as exercise [21]. Use of problem-focussed coping strategies such as planning was also consistently associated with more positive outcomes. On the other hand, emotion-focussed coping strategies including wishful thinking and escape/avoidance were consistently linked with worse adjustment [22,23]. High perceived levels of stress were also linked to worse adjustment [23]. Similarly, a tendency to interpret events, the illness and MS symptoms in an overly negative fashion has been linked to worse adjustment [24-26]. Since many of these factors are potentially modifiable through psychological intervention there is reason to conclude that a programme of therapy that addresses a number of these factors may lead to improvements in adjustment.

In the intervention literature to date targets of psychological interventions for people with MS, particularly Cognitive Behavioral Therapy (CBT), have been mostly centred on the alleviation of depression [27-29]. Two systematic reviews of psychological interventions for MS suggest that there is some evidence that cognitive behavioural approaches can be beneficial in the treatment of depression, and in helping people to adjust to, and cope with having MS[15,30] However, both reviews also criticise the methodological quality of the intervention studies to date. Many have small sample sizes and many inadequately report details of their intervention and trial methodology. This makes it hard to draw definitive conclusions about the efficacy of CBT in this area. Both reviews conclude that further, well-designed trials of CBT-based approaches are warranted. One in particular, concludes that existing research on adjustment and coping looks encouraging and that approaches that target the time period soon after diagnosis and see adjustment as an ongoing process would be useful [15]. This review also draws particular attention to the importance of following the CONSORT statement [31] for reporting such trials and using manuals to make replication of interventions possible.

### Background to the project

Given the evidence that CBT is helpful in improving mental health and psychological aspects of chronic health conditions, we wanted to investigate the efficacy of CBT in helping people with MS adjust to the psychological and social challenges of living with the disease. Although there are some differences between regional MS services in the United Kingdom in availability of information, advice and support in the early stages of MS, formal psychological interventions are not routinely available.

The aim of this project is to assess whether CBT can enhance the adjustment of people in the relatively early years of dealing with MS. Unlike most previous studies which have evaluated the use of CBT in this group [15,30], the focus of this study is to improve broadly defined adjustment outcomes rather than restricting the scope of the intervention to reducing psychiatric morbidity in MS populations where individuals meet criteria for psychiatric disorder. By focusing the intervention on the earlier years of MS it is hoped that patients will learn strategies that will assist in managing the illness in the future.

Because most MS patients receive little structured psychological support, CBT will be compared to Supportive Listening therapy rather than treatment as usual. This will allow us to ascertain whether there are any particular benefits to CBT or whether just having the opportunity to talk to a supportive, empathetic therapist can make a difference to adjustment outcomes. In line with suggestions for research in this area, general nurses will be trained and supervised to deliver the interventions [15].

### Aims

#### Primary Aim

1) To determine whether patients with early stage MS who undertake an 8 session CBT programme for adjustment to MS will demonstrate better adjustment (defined as psychological well-being and social and role adjustment) than those undergoing 8 sessions of Supportive Listening (SL).

#### Secondary Aims

2) To examine whether the CBT and SL groups differ on a range of secondary outcomes including quality of life, acceptance of illness and dysfunctional cognitions.

3) To examine whether changes in predefined psychological mechanisms act as mediators through which change/adjustment in our primary outcomes occurs. These proposed mediating factors are dysfunctional or unhelpful cognitions and behaviours, and acceptance of illness.

4) To examine whether patients' responses to treatment is moderated by therapeutic alliance factors, patients' treatment preference, engagement in homework tasks, and perceived social support.

5) To examine the cost-effectiveness of both interventions by taking into account benefits to patients, effects on health service usage and other costs to society, and cost of providing the interventions.

6) To evaluate both interventions from the perspective of the person with MS using in-depth interviews and qualitative analysis methods to elicit their experiences of the therapy, any changes it brought, and any helpful and unhelpful aspects.

## Methods/Design

### Design

**Figure 1:**
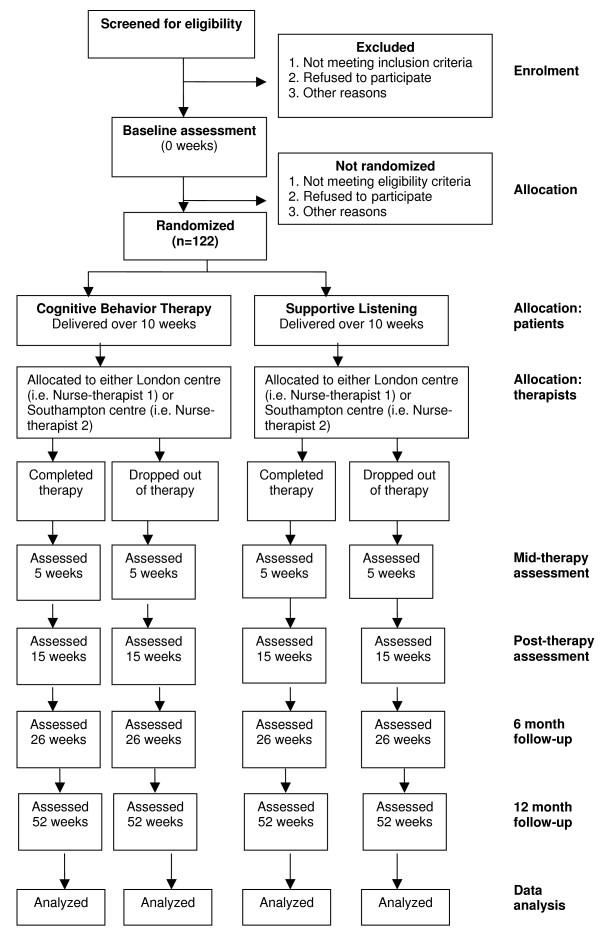
**CONSORT flowchart of trial design**.

The trial is a two arm randomized multi-centre parallel group controlled trial. Consenting participants who meet eligibility criteria are randomly allocated to receive either CBT for adjustment to MS or Supportive Listening (SL). The SL arm of the trial has been chosen over standard medical care as a comparison condition as it is an acceptable and plausible approach that will control for non-specific treatment factors such as support, therapist time and attention, and patient expectations of improvement. The full trial design is summarized in Figure 1.

### Setting

The trial has two centers (Southampton and South London) and each center has one therapist who delivers both CBT and SL.

### Ethical approval

This study has been reviewed and approved by the Thames Valley Multi-centre Research Ethics Committee (ref: 07/MRE12/6)

### Participants

#### Sample size calculation

Since there were no available MS CBT trials using either of our primary outcomes we computed a power analysis calculation based on a recent CBT trial for depression in people with MS, which used the Beck Depression Inventory (BDI) as the primary outcome [28]. The BDI, like the GHQ (one of our primary outcomes) is a measure of affective distress. Using a conservative estimate for the within-group standard deviation the effect size from this study was .60. To achieve 80% power at alpha = 0.025 significance level (to adjust for two primary outcomes) we would need 55 patients in each group to detect an effect of this magnitude by an independent samples t-test. To allow for attrition of around 10% at follow-up we need to initially include 61 participants in each group. In our CBT for MS fatigue trial we only lost 3/72 patients to follow-up (4%).

### Eligibility criteria

#### Inclusion criteria

Participants do not need to be currently experiencing distress, depression or any particular coping difficulties to be included in the study. However, they must:

- have a definite diagnosis of MS, of any type, confirmed by a neurologist.

- be diagnosed with MS within the last 10 years.

- have some degree of ambulation (with assistance if needed), equivalent to an Expanded Disability Status Scale (EDSS) score [32] of 6.5 or less.

- be stabilised on disease modifying or anti-depressant medication (if taking). For disease modifying drugs (e.g. interferon) patients must have been on medication for a minimum of 3 months. For anti-depressants, patients must be on a stable dose for a minimum of 2 months.

#### Exclusion criteria

Patients will be excluded from the study if they:

- have gross cognitive impairment that would make participation in 8 one hour sessions of talking therapy problematic or distressing. This will be assessed using the Telephone Interview for Cognitive Status-Modified; TICS-M [33] administered by the trial co-ordinator during screening. Patients with a score of less than 20 will be excluded.

- have serious psychological disorders for which treatment would be inappropriate (including psychotic disorders or active substance abuse problems).

- have other co-morbid serious chronic illnesses (e.g. a malignancy).

- are currently participating in other psychological therapies or have participated in other therapies within the last 2 months.

- are considered by their treating physician to have needs that are more appropriately addressed by a referral to another psychological service (e.g. psychiatrist, clinical psychologist, MS special mental health nurse).

#### Source of participants

Participants will be recruited from two National Health Service (NHS) MS centers in the Southampton area (Southampton University Hospital Trust; SUHT) and South London (King's College Hospital Trust; KCHT).

#### Recruitment and consent process

Patients seen or in contact with these services during the trial period will be informed about the trial if they appear to meet eligibility criteria. During routine consultations, either one of the MS nurses or one of the neurologists will use an eligibility checklist, detailing the key inclusion and exclusion criteria, to identify suitable patients. The nurse/neurologist will hand over a Participant Information Sheet with a contact details reply slip, consent form, and freepost envelope to all that are potentially eligible and interested. If the participant is keen to be involved the nurse/neurologist will ask permission to write down the participant's contact details and freepost it back to the trial co-ordinator so that she can contact them about the project. Alternatively the patient can choose to initiate contact with the research team by posting the contact details form back to the trial co-ordinator, or getting in touch by telephone or email.

Once contact is made with the trial co-ordinator the patient's questions will be answered and their understanding of what is involved in the trial verified. Consent forms will be returned to the trial co-ordinator by freepost for those who wish to take part. On the form, participants are given the choice to consent to the therapy trial and questionnaire assessments only, or to an additional in-depth telephone interview after their therapy programme has been completed.

#### Eligibility screening and enrolment

After obtaining written consent, participants will be screened for eligibility. Screening will be completed by the trial co-ordinator over the telephone using a screening checklist of the inclusion/exclusion criteria.

If potential participants do not meet the medication or current/recent psychological therapy criteria at the time of the eligibility screening they will be placed on hold and there will be the necessary delay (see inclusion/exclusion criteria) before they are enrolled into the trial. After screening, participants will be notified on the telephone by the trial co-ordinator whether they are eligible or ineligible for the trial.

#### Baseline questionnaire assessments

Eligible participants will be sent the baseline questionnaire and freepost return envelope together with a copy of their consent form. The schedule of assessments in the baseline questionnaire and subsequent follow-up questionnaires is outlined in Table 1.

**Table 1:** Details and schedule of saMS trial assessment procedures

Measures administered	Baseline (week 0)	Mid-therapy (week 5)	Post-therapy (week 15)	6 month follow-up (week 26)	12 month follow-up (week 52)
Demographics	X	-	-	-	-

Detailed MS questions-(date of diagnosis, time since symptoms started, type of MS, recent and current relapses, treatment for depression, supplements or alternative therapies, participation in MS organizations)	X	-	-	-	-

Brief MS questions (recent changes in medication, interventions, recent and current relapses)	-	-	X	X	X

Expectations/preferences for therapy	X	-	-	-	-

Global improvement and satisfaction ratings	-	-	X	-	-

Feedback on CBT manual/strategies	-	-	X	-	-

Continuation of use of CBT	-	-	-	X	X

General Health Questionnaire (GHQ-12 [41])	X	X	X	X	X

Work and Social Adjustment Scale (WSAS [43])	X	X	X	X	X

Beliefs about Emotions Scale (BES; Rimes & Chalder; in preparation)	X	X	X	X	X

Significant Others Scale (SOS [49])	X	-	X	X	X

Acceptance of Chronic Health Conditions Scale (ACHC [45])	X	X	X	X	X

Psychological Vulnerability Scale (PVS [46])	X	X	X	X	X

Brief Illness Perception Questionnaire (B-IPQ [48])	X	X	X	X	X

Cognitive and Behavioural Responses to Symptoms Questionnaire (CBRSQ; Moss-Morris, Chalder, Skerrett & Baldwin, in preparation)	X	X	X	X	X

Client Service Receipt Inventory (CSRI [51])	X	-	-	X	X

EuroQoL (EQ5D [44])	X	-	-	X	X

Self-report Expanded Disability Status Scale (EDSS [50])	X	-	-	-	X

#### Randomization

Randomization is at the patient level and will take place after the baseline questionnaire has been received. The randomization will be stratified by centre to ensure equal proportions in each treatment arm. Randomization is block stratified with varying block sizes. Randomization will be handled by an independent service at the King's College Mental Health and Neuroscience Clinical Trials Unit (CTU). On receipt of the baseline questionnaire, the trial co-ordinator will electronically submit details of each participant to an electronic randomization system set up and maintained by the CTU. The system then notifies the relevant nurse-therapist (i.e. London or Southampton) of the randomisation outcome by email immediately. This email will be printed and placed on the participant's therapy file.

#### Booking in participants

On receiving the randomisation outcome from the CTU the nurse-therapist will contact the new participant, notify them of their group allocation and set a date for their first session. Nurse-therapists will endeavour to book patients within one week of randomisation. Participants will be consulted about special considerations needed for their visit (e.g. disabled parking) and reimbursed for their travel expenses.

#### Therapist training

The therapists in this trial are two general nurses without prior experience in delivering psychological interventions, or specialist MS experience. They were given 2 weeks of intensive classroom training in understanding MS, CBT and SL.

The training included a combination of didactic teaching, homework readings, and discussion. Role play and videoed therapy sessions were also used throughout the CBT and SL training sessions. A summary of the classroom training package is presented in Table 2.

**Table 2:** Details of the two week classroom training program for the nurse-therapists

Pragmatics of the trial and house keeping (6 hours)	• Line management and orientation to the units
	• Record keeping for the trial, general paperwork, keeping patient notes and writing GP letters
**Understanding MS: (6.5 hours)**	• Introduction to MS including the pathogenesis of the disease, types of MS, symptoms and medical treatment
	• Understanding adjustment to MS and introduction to the CBT model and manual
	• The role of partners and family members

**Cognitive Behavioral Therapy (32.5 hours)**	• Introduction to Beck's systems model of CBT
	• Introduction to CBT, psychological therapies, and psychiatric diagnosis
	• Process of treatment and how change takes place over time
	• Assessment, conceptualization and formulation in CBT
	• Supervision and how to use it
	• Exploring the nurse-therapists' personal beliefs and assumptions
	• Motivational Interviewing Skills
	• Setting homework
	• Overview of anxiety and depression, including prevalence and diagnostic criteria.
	• Emotional processing and how to facilitate it.
	• Psychiatric emergencies and risk assessment
	• Identifying MS specific cognitions
	• How to use thought records to link, thoughts, behaviors and emotions
	• Challenging unhelpful thoughts
	• How to deal with patients "realistic" negative thoughts
	• Behavioral Activation and behavioral experiments
	• Problem solving
	• Dealing with resistance
	• Dealing with hopelessness

**Supportive Listening (13 hours)**	• Introduction to therapist and patient manuals
	• Issues around housekeeping
	• Introduction to concepts of unconditional regard, warmth and empathy
	• Describing the active listening techniques
	• Facilitator demonstration of techniques
	• Nurse role play using MS case studies
	• Nurse role play using a personally relevant difficult or painful situation
	• Practice and feedback using the variety of techniques

The classroom training was followed by 12 weeks of closely supervised practice with 5 pilot patients. Each nurse saw 3 pilot patients for 8 weeks of CBT and 2 pilot patients for 8 weeks of Supportive Listening. These patients had a diagnosis of MS but did not necessarily meet other trial criteria. RMM supervised the SL and TC and SR supervised the CBT.

Supervision of pilot patients occurred once a week as soon after a session as possible. The supervisor listened to one of the recent sessions and rated the session using the Therapy Competence and Fidelity Rating scale modified specifically for this trial [34] This scale includes 14 items, 7 of which assess SL and 7 CBT. Example items are 'Does the therapist reflect or paraphrase appropriately' (SL) and 'Does the therapist help the client to identify specific types of cognitive distortions or errors (e.g. all-or-nothing thinking, over-generalization) (CBT item). The nurse-therapist also listened to the session and completed self-ratings of competence using the same rating scale. Supervisor and nurse ratings were discussed during supervision and strengths highlighted. Areas for improvements and strategies for these were also identified. Checks were made for treatment fidelity and to assess that CBT techniques were not used in the SL sessions. Approximately midway in the pilot phase of the training, both supervisors and nurses met to discuss pilot patients and any difficulties encountered. Difficult situations were role played and new skills or ways of managing the situations were practiced.

#### Establishing Therapist competence

During the pilot/training stage the nurse-therapists audiotaped every therapy session using digital recorders. The clinical supervisors rated two randomly selected recordings (sessions 5, 6, 7 or 8) using the Therapy Competence and Fidelity Rating scale described above. The therapists were required to score at least 4 (i.e. a 'acceptable-good' level of competence demonstrated) on all the relevant items in order to be deemed competent in the therapy and ready to commence therapy with trial participants.

#### Trial interventions

Both therapies will be delivered in 8 sessions over a 10 week period. The first six sessions will be scheduled weekly and the last two fortnightly. The first session will be 80–90 minutes long and the remainder will be between 50 minutes to one hour. The first and fourth sessions will be held face-to-face and the remaining six will be telephone sessions. Telephone sessions make CBT more accessible to patients, particularly those with mobility problems, and have been shown to be an effective form of therapy for people with MS [27].

Both interventions will be carried out in accordance with written, structured manuals. Participants will be issued either a CBT or SL therapy manual in their first session with the nurse-therapist.

### CBT

#### Development of the manual and therapy package

Beck's cognitive model of emotion which incorporates a developmental perspective [35] in conjunction with a systematic review of 72 studies which looked at psychological factors associated with adjustment in MS [16] was used to guide the development of the CBT model and therapy manual for this trial. Details of our CBT model of adjustment to MS are presented in the review [16]. Because acceptance of MS appeared to be a significant factor in adjusting to the illness, we incorporated some basic principles for facilitating acceptance into our manual from Steven Hayes' Acceptance and Commitment Therapy [36].

We also conducted two qualitative studies where 30 people with early stage MS and 15 of their partners engaged in in-depth interviews about their experiences of having MS, problems they encountered, and the things they found helpful and unhelpful in adjusting to living with MS [[37]; Dennison, Yardley, Devereux and Moss-Morris, in preparation]. Transcripts of these interviews were analysed using thematic analysis. Core themes were covered in the manual and verbatim quotes from the interviews were used for illustrating points.

Once the 100 page draft manual was developed, it was reviewed by people with MS and their families, neurologists, MS nurses and CBT therapists. Feedback from these sources was then used to make appropriate amendments to the manual.

We also developed a short 10 page booklet for the participant to give to a partner or significant other entitled "Coping when somebody close to you has MS". This booklet covers information which closely reflects the themes from the qualitative study on spouses of people with MS [37]. These include dealing with feelings of helplessness and difficult emotions, how to support someone with MS, dealing with other people's reactions to MS, maintaining a social life in the face of MS, and finding a balance between looking after your own well-being and the needs of your partner with MS.

#### The CBT therapy package

The aim of this CBT package is to enable patients to adapt appropriately to their illness. The focus is to achieve optimal day-to-day functioning within the constraints of the disease, to minimize distress and manage symptoms in the short and long term. The treatment is structured but also individualized to the needs of the patient as it is clear from the literature and our qualitative interviews that the process of adjustment can vary across individuals.

In the early sessions, participants will work with their nurse-therapist in developing a formulation of their particular areas of strengths and difficulties. The manual consists of nine chapters which can be used as appropriate depending on the formulation (see Table 3 for details of the manualized sessions). The nurse-therapist will work together with the participant in deciding which areas to focus on and in setting tasks or homework to do in between the sessions. The nurses will record which of the nine chapters are covered in each session for each patient. Both nurses and patients will work from the same detailed manual.

**Table 3:** Summary of the content of the CBT manual for adjustment to MS

Chapter 1: Introduction to adjusting to MS	What is MS and what does adjusting to MS mean?
	Factors which have been shown to affect adjustment in MS.
	A CBT model of adjusting to MS which includes interactions between thoughts, behavior, biology and emotions.
	Assessment of current strengths and difficulties.
**Chapter 2:** Adapting to living with MS	What do we mean by acceptance?
	Strategies for becoming accepting.
	Dealing with negative emotions such as sadness, grief, loss, frustration, anger, anxiety, depression, shame and embarrassment.

**Chapter 3.** Setting goals and problem solving	Exploring values and setting treatment goals where change may be needed across different areas of life.
	Once problems are identified, developing a stepped approach to problem solving drawing on the patient's strengths and support network.

**Chapter 4.** Managing symptoms	Helping make the link between symptoms, thoughts and behaviors. May involve a discussion on accepting limitations.
	The pitfalls of becoming overly symptom focused and avoidant and strategies for managing these.
	Understanding MS symptoms, and which symptoms are likely to be a sign of relapse, medication side effects, or stress/distress.
	Diaries of patterns of rest and activity to see how these may influence symptom experience.

**Chapter 5.** How to tackle negative and unhelpful thoughts	Demonstrations of how perceptions of events can influence coping with illness.
	Identifying traps or 'errors' in thinking and finding alternatives can help with adjustment and levels of distress.
	Examples of unhelpful thoughts are covered such as fears about the illness and future, and high personal expectations.
	Using daily thought records of unhelpful thoughts, challenging these thoughts and coming up with alternative thoughts.

**Chapter 6.** Improving the quality of your sleep	Basic sleep hygiene including establishing a good sleep/wake routine which encourages natural sleep and addressing factors which interrupt sleep.
	Goal setting to improve sleep.

**Chapter 7.** Managing stress	Exploring skills to call on in times of stress such as distraction, problem solving, relaxation, prioritising, saying no and planning.
	Goal setting to improve stress management.

**Chapter 8.** Managing social relationships	Becoming more assertive.
	Managing relationships with care providers.
	Getting the right type of support for one's needs and sharing emotions.

**Chapter 9.** Preparing for the future	Identifying physical and emotional warning signs of relapse and normalising these.
	Developing a future management plan using personal strengths, newly learnt skills and support from others in difficult times.

#### Supportive Listening

Supportive Listening (SL) will be presented to patients as a treatment based on the idea that they will be able to help themselves if given the opportunity to talk freely, extensively and confidentially about their experiences, thoughts and feeling about MS and its effect on their lives. If participants prefer not to focus on their MS, they will be encouraged to choose other topics to talk about which, they feel, are currently relevant to them.

The SL therapist manual is based on manuals used in previous trials comparing CBT to counselling [38] and pragmatic rehabilitation to Supportive Listening therapy [39]. The manual outlines how SL is different to CBT and which interventions are prohibited such as offering explanations for symptoms, eliciting symptom information or changes, suggesting explicit coping strategies, interpreting information rather than responding reflectively, leading or directing the client and giving homework assignments or tasks.

The SL therapist manual also includes a description of the listening skills to be used in SL which are based on the theories and counselling techniques of Carl Rogers [40]. These core skills include asking open questions, active listening skills such as minimal encouragers, paraphrasing, empathising, reflecting and summarising. The purpose is to provide the participant the opportunity to talk and express themselves in a non-judgemental, safe environment. The person should experience empathy from the therapist and feel listened to.

More advanced Rogerian counselling skills such as problem clarification, accurate understanding and challenging are not included in the therapist training or manual. The SL was designed to control for the non-specific effects of therapy such as warmth and positive regard rather than being formal counselling therapy per se.

The participant or client manual for SL is a short 7 page document. It provides brief information about MS and why Supportive Listening may be beneficial for people with MS. It also outlines what patients can expect from the therapy sessions and a timetable for scheduling the meetings.

#### Missed or postponed sessions

In the case of cancellation or non-attendance at therapy the nurse-therapist will contact the participant by telephone to ascertain the problem of attendance and will discuss an appropriate solution. If the session can be rearranged to stay in line with the standard scheduling of sessions it should be rescheduled.

Inevitably there will be times when MS symptoms or relapse, other sickness, holidays, or unexpected events interfere with the standard scheduling of sessions such that therapy exceeds or alters the 10 week window (described earlier). However, generally therapy should be completed within a maximum of 12 weeks. In exceptional circumstances (e.g. the participant experiences a severe relapse of MS or has a family/personal crisis) timing of sessions can be discussed with principal investigators and rearranged. However, a participant will never have more than the 8 sessions.

#### Therapy record keeping

Deviations from the standard therapy schedule will be logged so they can be reported and analysed. Details of the date and length of each session (and for CBT participants, the chapters covered and a rating on a scale of 1–10 of homework engagement) will be recorded by the nurse-therapist.

All therapy sessions will be digitally recorded with permission from the participant and saved in an encrypted anonymous format. These recordings will be used for ongoing supervision and to assess treatment fidelity and therapist effects at the end of the trial.

#### Informing the participants' health professionals regarding involvement in the trial

After the first session, the nurse-therapist will write to the participant's GP and MS service to inform them of the patient's participation in the trial. For CBT patients this will also include a summary of the assessment made and the areas that are likely to be concentrated on in the remaining sessions (discussed and agreed with the participant and a copy sent to the participant for their own records).

#### Ongoing supervision of nurse-therapists

Two experienced CBT therapists (SR and TC) will supervise the CBT and an experienced health psychologist (RM) will supervise the SL. For the first two months of the trial each nurse-therapist will have a separate CBT and SL supervision session once per week. Supervision will be fortnightly for the rest of the trial. Sessions will be between 30 – 60 minutes long and may be either by telephone, or face-to-face. At least once every two months the nurse-therapists will have a face-to-face session. Tape recordings of a session will be shared with the supervisor before the sessions and discussed during supervision. Each nurse-therapist will keep a log of dates and timings of their supervision sessions. Once per month the supervisor will check that session documentation has been properly completed.

#### Therapeutic fidelity ratings

Fidelity rating for the interventions will occur throughout the trial in the supervision sessions. In addition, at the end of the trial a selection of recordings of therapy sessions will be assessed by an independent experienced clinician using a modified version of the therapy rating scale [34] to ensure treatment integrity. Sessions to be rated will be selected at the end of the therapy part of the trial without input from nurses and supervisors. Approximately one third (randomly chosen) will be co-rated by a second rater in order to establish reliability of ratings.

#### Outcome assessments

Questionnaires (see Table 1) will be completed at baseline (prior to randomisation), mid-therapy (session 5 of therapy sessions), post-therapy follow-up (15 weeks after randomisation), 6 month follow-up (26 weeks after randomisation) and 12 month follow-up (52 weeks after randomisation). All assessments are questionnaire-based. Data collection will be managed by the trial co-ordinator who will be blind to treatment allocation. Participants will be able to choose whether to complete online or paper-based questionnaires (returned by freepost). Dates that questionnaires are sent out and received will be recorded. A reminder phone call or reminder emails will be used if the questionnaire is not received within two weeks of send-out. After four weeks another copy of the questionnaire pack will be reissued.

If participants are unable to complete questionnaires themselves (e.g. due to relapse) the trial co-ordinator will go through the questionnaire on the telephone and this will be logged as such on the trial database. Spouses/partners/friends will not be allowed to complete the questionnaire on the participant's behalf or assist them with writing their answers. Completed, returned questionnaires will be checked by the trial co-ordinator and participants contacted if there is a large amount of missing data.

### Primary outcome measures

There are two primary outcomes.

1. The General Health Questionnaire (GHQ) [41] is designed to measure general levels of distress in people in the community and medical settings. The measure is uncontaminated by the experience of MS related somatic symptoms and a recent study showed the GHQ was the most treatment responsive measure of psychological distress in three discrete MS samples [42].

2. The Work and Social Adjustment Scale (WSAS) [43] is a self-report scale of functional impairment attributable to an identified illness, in this case MS. The scale measures how much MS interferes with a person's work, home management, social leisure activities, private leisure activities, and the ability to form and maintain close relationships.

### Secondary outcome measures

These include quality of life and factors which have been shown to be related to adjustment outcome in MS and are key components of our model of adjustment to MS.

1. Quality of life will be measured using the EuroQol 5 (EQ-5D) [44] which is a 5-item composite measure of mobility, self-care, usual activities, pain/discomfort and anxiety/depression.

2. Acceptance of illness will be measured using the Acceptance of Chronic Health Conditions Scale (ACHC) [45]. This scale assesses acceptance of and adjustment to change in one's life due to a chronic health condition (in this case MS).

3. Dysfunctional cognitions will be measured by the Psychological Vulnerability Scale (PVS) [46]. This short scale taps maladaptive cognitive responses which are proposed to promote unhelpful adjustment to stressors (e.g. perfectionism, need for approval).

#### Mediators of the treatment effect

In order to gain a clearer idea of the possible mechanisms and to help refine our CBT model of MS adjustment we have included a number of measures as possible mediators of the treatment effect. These are cognitive behavioural factors which have been demonstrated to be associated with adjustment outcomes, and/or are addressed within either or both interventions. We hypothesize that the mediators of improvements in psychological well-being (GHQ) will be different to the mediators of improvements in functional impairment (WSAS).

#### Mediators of the WSAS

1. Cognitive and behavioural responses to symptoms. The Cognitive and Behavioural Response to Symptoms Questionnaire (CBRSQ) [Moss-Morris, Chalder, Skerrett & Baldwin, in preparation] is a newly devised 34 item questionnaire which was designed to measure patients' cognitive and behavioural responses to symptoms. The subscales of this questionnaire have been shown to predict a significant amount of the variance in MS related disability and fatigue over and above EDSS scores, remission status, and mood [47]

2. Illness perceptions will be assessed using the Brief Illness Perceptions Questionnaire (BIPQ) [48]. This 8 item questionnaire assesses cognitive and emotional illness representations (e.g. its timeline, consequences, controllability). Across a range of illnesses, negative illness perceptions are related to worse adjustment outcomes, whilst changes to illness perceptions may improve adjustment.

#### Mediators for GHQ

1. Unhelpful beliefs about emotions are measured using the recently developed Beliefs About Emotions Scale (BES) [Rimes & Chalder; in preparation]. This 6 item questionnaire measures the extent to which the person holds unhelpful beliefs about experiencing, expressing, and controlling emotions.

2. Dysfunctional cognitions (PVS)[46] as described above under secondary outcomes.

3. Acceptance of illness (ACHC) [45]as described above.

#### Moderators of Treatment Improvement

We will also look at two potential moderators of patients' response to treatment, the therapeutic alliance and perceived social support.

The therapeutic alliance is measured using the Alliance subscale of the Therapy Competence and Fidelity Rating scale [34]. Independent raters blind to treatment outcome will use this scale to rate therapy tapes from each of the two therapists. The scale includes items including emotional expression by the patient and level of empathy expressed by the therapist. Items are rated on a 7-point Likert Rating Scale, with anchors at four points along the scale [e.g. 'not at all' (1), 'somewhat' (3), 'considerably' (5) and 'extensively'].

Social support is measured by the Significant Other Scale (SOS) [49]. We are using a shortened version of this scale (8 items) where the participant is asked to state one key support person and then rate desired and received support in different domains (e.g. practical help and socialising).

In addition to these measures we have included a number of questions to measure illness severity, progression and relapse (see Table 1) including the self-reported Expanded Disability Status Scale (EDSS)[50]. This will allow us to assess whether there has been any noticeable disease progression. The self-reported EDSS is a relatively new instrument which allows MS patients to self-report their current disease status, rather than this being assessed by a neurologist during a clinical examination. The questionnaire includes items which relate to mobility, strength, co-ordination, sensation, bladder, vision, speech, swallowing, and cognition. Using the responses from these items, functional system scores are computed and an EDSS score is assigned to the participant ranging from 0 (no neurological impairment) to 10 (death from MS). The trial co-ordinator will review and score each questionnaire, which is then co-scored by a neurologist specialising in MS in order to ensure reliability of scoring.

Patients will also be asked to rate their treatment preference at the beginning of the trial and their feelings about treatment efficacy at the end of the trial.

### Service use and QALYs

Service use (additional to the CBT intervention or Supportive Listening which will both be centrally recorded) by patients in both arms of the trial will be measured using the Client Service Receipt Inventory (CSRI) [51]. This will record health and social service contacts in the six months prior to baseline assessment, and the periods prior to 6- and 12-month follow-up interviews. Time lost from work because of MS-related problems will also be measured.

The EuroQol (EQ-5D) [44], a 5 item composite measure which considers mobility, self-care, usual activities, pain/discomfort and anxiety/depression will be used to assess quality of life. The EQ-5D will be used to generate quality adjusted Life Years (QALYs) [52].

#### Blinding

The trial co-ordinator will administer all quantitative assessment procedures and will be kept blind to the treatment allocation of participants until final follow-up has been completed. The trial co-ordinator will not deal with paperwork that links a participant to their therapy condition, or be present when any discussions revealing this information take place. However, if she becomes aware or suspicious of the treatment condition of any participant she will log this lapse of blinding on the trial database for later consideration in a sensitivity analysis. Either way, as all assessments are by self-report, rather than rated by a member of the research team, influences of observer biases are not expected.

Due to the nature of the interventions it is not possible to blind the principal investigators and nurse-therapists as they will be delivering and supervising the therapy sessions. It is also not possible to blind participants to treatment allocation. However, the trial is set up as comparison of two treatments rather than as a treatment versus control condition. The participant information presents both treatments as a way of assisting adjustment to MS with neither treatment being given preference. During statistical analysis the treatment groups will be assigned a code rather than their name so that the statisticians are unaware what the treatment group code represents.

### Data inputting

Data will be input into a flat SPSS spreadsheet by the trial co-ordinator. All primary data (GHQ and WSAS) at all time points will be double entered by an administrator. This will then be checked for errors and discrepancies, which will then be corrected by consulting the raw questionnaire data (paper based or online). A percentage of all other data inputting will also be double entered to ensure accuracy. Any follow-up action (e.g. double entry of more data) will be taken as necessary.

#### Qualitative interviews

On receipt of completed post-therapy questionnaires, details of those participants who have consented to take part in the in-depth interview will be passed to a post-doctoral researcher who has not had any involvement in other aspects of the trial.

Around 15 CBT participants and 15 SL participants will be interviewed regarding their experiences of therapy. If a large enough pool of consenting participants is available, purposive sampling will be employed on the basis of questionnaire-based reports of therapy satisfaction, perceived improvement, and demographic and illness characteristics.

The interviewer will contact each participant to schedule a telephone interview and conduct the interview according to an interview schedule. Questions address expectations of therapy, experiences of therapy sessions, features of therapy liked and disliked, and the process of change (or not) as a result of therapy. Each interview will be audio-taped or digitally recorded. Interviews will be transcribed by an administrator, omitting any information that may compromise confidentiality. Completed recordings and transcripts will be stored in a locked cabinet (if printed) or in a password protected file (if digital) for analysis after the trial has concluded.

#### Independent monitoring and quality control

A Trial Steering Committee (TSC) including a Data Monitoring Group has been set up to monitor the conduct of the trial. They will provide overall supervision for the trial and safeguard its integrity. Authority for continuation of the trial lies with the TSC. The committee includes an independent chairperson, the lead and principal investigators, the trial co-ordinator, a statistician, a representative from the MS Society and a patient representative. The committee will meet at least annually during the trial period, and more frequently and/or via telephone conference or email communication if pressing issues arise.

#### Compliance

The trial will be conducted in compliance with the Declaration of Helsinki, MRC Good Clinical Practice (GCP) guidance, the Data Protection Act (1998), the National Research Ethics Service (NRES) approvals, NHS Trust regulations, and other regulatory requirements as appropriate. The final trial publication will include the items recommended under the extended CONSORT statement for randomized trials of nonpharmacologic treatment [53].

#### Participant safety

Both CBT and SL are expected to be of low risk to participants. They are non-invasive talking therapies and similar interventions have been previously used in chronic illness populations. If the nurses are concerned about a patient's safety, particularly if they believe the person may be vulnerable to self-harm, they immediately contact one of the therapy supervisors who will telephone the patients to assess the severity of the situation. If necessary, a referral will be made to ensure the patient gets the support they need.

#### Adverse events/reactions

Adverse events (AE), adverse reactions (AR), serious adverse events (SAE) and serious adverse reactions (SAR) will be defined according to the usual clinical trial definitions. Where these occur they will be recorded on the trial spreadsheet and reported to the appropriate authorities and followed up in the standard manner.

#### Stopping/discontinuation rules

Departures from the trial protocol, changes to the manualized treatments, and breaking of the randomization code will only occur with the advice of the TSC if it becomes apparent that a particular treatment arm is causing a consistent pattern of deterioration, or if there is another obvious and significant clinical necessity.

#### Withdrawals from therapy

If a participant expresses the wish to withdraw from the trial, the nurse-therapist will contact the participant to ascertain the reason for drop-out if the participant is willing to share this. They will be offered the option of talking to a principal investigator instead if they wish. If the participant considers that they are deteriorating a principal investigator will contact them and discuss an appropriate solution.

The nurse-therapist (or principal investigator if they make contact) should ascertain whether consent is withdrawn from:

a) further treatment only

b) further treatment and follow-up (questionnaires plus the in-depth telephone interview)

c) retaining data already collected for use in final analysis.

The reason for withdrawal (e.g. adverse events, relapse, illness progression, inability to adhere, inability to attend) will be communicated to the principal investigator for that centre as well as the trial co-ordinator who will record and report this information as appropriate.

If it is felt that a participant should be withdrawn from the trial this will be discussed with the nurse-therapist, principal investigator and clinical supervisor. One of the principal investigators will assess the participant clinically within a week and arrange appropriate ongoing care. The nurse-therapist will write to the participant's GP and MS service to confirm withdrawal from treatment.

#### Statistical analysis plan

Statistical analysis will be carried out in SPSS and/or Stata general purpose statistical analysis packages. Mediation modeling may require use of specialist structural equation modeling software (e.g. AMOS or M-Plus).

All treatment group comparisons will be carried out on an intention-to-treat basis, that is subjects will be analyzed in the group to which they were randomized irrespectively of the treatment received. The primary and secondary longitudinal outcomes will be analyzed using linear mixed modeling. In these models the outcome variable at the post-treatment time points will be the dependent variable and baseline values of the outcome variable, centre, time (post-treatment, 6 M follow-up or 12 M follow-up), group (CBT or Supportive Listening) and a time × group interaction terms will feature as explanatory variables. To account for correlation between measures taken on the same individual at various time points subject-varying random intercepts and slopes of time will also be included in the model. Further baseline variables might be used as explanatory variables, to provide more powerful group comparisons if they are found to be predictive of the outcome variable. As the model fitting will by maximum likelihood such analyses are valid if missing data arises at random (MAR). The effect of informative missingness processes will be explored by means of a formal sensitivity analysis [54].

We will further investigate whether results were unduly influenced by patients who have shown marked disease progression during the trial or who have altered medication during the trial period. We will also assess the sensitivity of the results to excluding patients who did not receive a sufficient number of treatment sessions or those who had low expectations of the therapy they received.

An approach similar to Baron and Kenny's mediation modeling [55]will be used to explore whether change of psychological outcomes mediates the path from therapy to change in MS adjustment. Specifically we will use instrumental variable methods advocated by Dunn and colleagues to try and adjust for unobserved confounders of the path from mediations to outcomes [56,57].

### Economic evaluation plan

Service use measured with the CSRI will be combined with relevant unit costs (e.g. [58]). The cost of the interventions will be calculated using information on therapist pay as well as training and supervision costs and overheads. Cost comparisons will be made between the two groups using bootstrapping methods to account for non normality in the data distribution.

Service cost data will be combined with the primary outcome measures (GHQ and WSAS) and quality-adjusted life years (QALYs), generated from the EQ-5D to assess cost-effectiveness. If the intervention has lower costs and better outcomes then it will be 'dominant'. In the event of the intervention having higher service costs and better outcomes, cost-effectiveness will be assessed using incremental cost-effectiveness ratios. To address uncertainty in cost and outcome differences we will use cost-effectiveness planes (CEPs) which will show the probability of the intervention being (i) cost-saving and more effective, (ii) cost-saving and less effective, (iii) cost-increasing and more effective and (iv) cost-increasing and less effective. Cost-effectiveness acceptability curves (CEACs) will be generated to show the probability that the intervention is cost-effective for different values placed on a unit improvement in the GHQ/WSAS or one extra QALY gained. CEPs will involve producing a large number of cost-outcome combinations using bootstrap methods and plotting these on a plane where one axis represents incremental costs and the other incremental outcome. CEACs will be generated by computing 'net benefits' for each participant (defined as the monetised value of outcome minus service costs). The value of a unit improvement in outcome is unknown and therefore a range of values will be used resulting in a number of different net benefits for each participant. Regression analyses will be used to estimate the difference in net benefits between the two arms for each value placed on a unit improvement in outcome. Bootstrapped regression coefficients of these differences will be saved and the proportion that are above zero will indicate the probability that one arm is more cost-effective than the other.

#### Qualitative analysis plan

A thematic analysis of the participants' therapy experience interviews will be conducted based upon procedures described by Boyatzis and Joffe and Yardley [59,60]. This will involve an inductive approach whereby transcripts are coded in order to develop conceptual categories which describe salient themes in the data. Elements of grounded theory practice [61,62] will also be incorporated; constant comparison, "in vivo" coding, attention to discrepant cases and memoing. Once all transcripts are coded and a comprehensive coding manual has been developed and refined we will inspect the data for patterns and relationships in the themes within and between the two therapy groups.

#### Reporting and Dissemination

A number of publications are expected from this trial including:

• Main outcome paper

• Economic analysis-costs/benefits

• Qualitative analysis of patient feedback and experiences

• Predictors of treatment outcome

• Mechanisms of change during therapy

• Cross-sectional study of factors involved in adjustment from baseline data

We expect to present the findings at various scientific forums including MS specific and neurology conferences, health and clinical psychology conferences, and behavioural medicine meetings. We will also report back to our funder the MS Society and patient led meetings such MS Life. A lay summary of the results will be sent to trial participants.

#### Study status

The trial opened to recruitment in January 2008. Enrolment and the therapy interventions will continue until early 2009. Throughout 2009 follow-up assessments of participants will be conducted. Results will be analyzed and reported in 2010.

## Discussion

To our knowledge this is the first RCT to look specifically at assisting with broadly defined psychological adjustment in the early stages of the MS. The relatively inclusive eligibility criteria for this trial mean that we are offering therapy to a group of patients who would not otherwise routinely have any formal psychosocial interventions, but who nonetheless are faced with considerable adjustment challenges.

This trial will answer important questions about the efficacy of an 8 session manualized CBT treatment in reducing distress and MS-related social and role impairment as well as a number of secondary outcomes. It has a relatively long follow-up period (12 months) which allows for the assessment of both short and longer-term effects of the interventions. The trial will also provide important data regarding the cost-effectiveness of both interventions and their acceptability to patients. The trial is also set up in such a way to study mediators of change during therapy and therefore shed light on the process of adjustment and the important factors involved.

If the CBT (or indeed the SL) is an efficacious and cost-effective intervention for assisting with adjustment to MS, the fact that it has been manualized and the details of training, supervision and therapy delivery are intricately described means that it should be straightforward to replicate on a larger scale. The use of general nurses as therapists in this trial is a significant advantage. If therapy can be successfully learned and delivered by general nurses with no prior experience of psychological interventions, it suggests that it would not be necessary to employ health professionals with high-level training in clinical psychology (who are in short supply and expensive to services) to deliver adjustment interventions to those with MS. Indeed, they could potentially be offered by health professionals who are already working with MS patients in the NHS (e.g. specialist MS nurses and occupational therapists). Furthermore, the use of telephone contact for the majority of the therapy sessions means that the intervention could be delivered in a flexible way that could reach more patients. Mobility difficulties and other disabling and unpredictable symptoms might be significant barriers for people with MS accessing face-to-face services.

The trial is set up to be methodologically robust and to conform to best practice for the conduct and reporting of RCTs. We have taken care to address key sources of bias; the manualized therapies are subject to ongoing supervision to ensure therapy fidelity and this will also be checked post-therapy by independent raters. The assessments of patient outcomes are self-reported and collected by the trial co-ordinator who is blind to treatment allocation and uninvolved in therapy delivery. The CBT is being exposed to a stringent test of it's efficacy since it is being compared to a potentially therapeutic intervention, SL. Although the SL is designed to control for non-specific therapeutic effects such as of the considerable time and attention from an interested and caring health professional, it is based on principles of counseling which allows patients to explore and work through issues is a non-directive format.

The saMS trial benefits from patient user input from the early stages of therapy design and manual development, right through to the in depth evaluation of the therapy sessions. The use of qualitative methods alongside the RCT means that the voice of those who would be using the intervention has been considered during both the design and evaluation of the therapy. This qualitative data will be particularly important in guiding any changes to the therapy format.

## Competing interests

The authors declare that they have no competing interests.

## Authors' contributions

RM and TC were involved in the conception and design of the study and applied for funding. RM wrote the initial grant, is the lead investigator and the Principal Investigator for the Southampton site, supervises the SL therapy and led the write up of the protocol. TC is the Principal Investigator for the London site. LY developed the idea for and supervised the qualitative studies and assisted in the initial grant application. LD helped develop and refine the study protocol, co-ordinates the trial, and has had significant input into the write-up of the protocol. SR contributed to the training and competency assessment of the therapists, conducts ongoing supervision of the CBT therapy and contributed to these sections of the manuscript .SL developed the statistical analysis plan. PM developed the economic analysis plan. All authors read and approved the final version of the manuscript.

## Pre-publication history

The pre-publication history for this paper can be accessed here:

http://www.biomedcentral.com/1471-2377/9/45/prepub
